# Social determinants of mortality due to visceral leishmaniasis in Brazil (2001-2015): an ecological study

**DOI:** 10.1590/0037-8682-0262-2019

**Published:** 2019-12-20

**Authors:** Bruno Eduardo Bastos Rolim Nunes, Thiago Cavalcanti Leal, João Paulo Silva de Paiva, Leonardo Feitosa da Silva, Rodrigo Feliciano do Carmo, Michael Ferreira Machado, Maria Deysiane Porto de Araújo, Victor Santana Santos, Carlos Dornels Freire de Souza

**Affiliations:** 1 Universidade Federal de Alagoas, Departamento de Medicina, Arapiraca, AL, Brasil.; 2 Universidade Federal do Vale do São Francisco, Colegiado de Ciências Farmacêuticas, Petrolina, PE, Brasil.; 3 Universidade Federal de Alagoas, Núcleo de Epidemiologia e Saúde Pública. Arapiraca, AL, Brasil.

**Keywords:** Leishmaniasis, Mortality, Epidemiology, Brazil

## Abstract

**INTRODUCTION:**

We aimed to analyze the relationship between visceral leishmaniasis mortality and social determinants of health (SDH).

**METHODS:**

This was an ecological study of all leishmaniasis-related deaths in Brazil, from 2001 to 2015. We analyzed 49 indicators of human development and social vulnerability. The association was tested using the classical and spatial regression model.

**RESULTS:**

Mortality was associated with indicators that expressed low human development and high social vulnerability: lack of garbage collection, low schooling, unemployment rate, low per capita income, and income inequality (Gini index).

**CONCLUSIONS:**

There was an association between high mortality by leishmaniasis and low SDH.

Leishmaniasis is among the neglected tropical diseases caused by protozoan parasites of the *Leishmania spp*., which are transmitted between humans and other mammalian hosts by *Phlebotomine* sand flies^1^. Depending on the species of *Leishmania* parasites and other immunological and epidemiological aspects, *Leishmania* infection can lead to cutaneous (CL), mucocutaneous (MCL), or visceral leishmaniasis (VL)^1^.

An increase in the number of cases of these diseases has been observed worldwide over the past decades, with an estimated 700,000 to 1 million new cases and 20,000 to 30,000 deaths annually, especially in middle- and low-income countries^1^. Of the 200 countries reporting cases of leishmaniasis, 87 are considered to be endemic^1^.

In 2016, Brazil, Peru, and Colombia accounted for 15% of all cases of CL worldwide^1^. In the same year, 12,690 new cases were recorded in Brazil, with an incidence rate of 13.0 cases per 100,000 population^2^. With regard to VL, Brazil, India, Sudan, and South Sudan accounted for 78% of all global cases in 2016^1^. In Brazil alone, 3,127 cases were reported, with an incidence rate of 1.5 cases per 100,000 population. VL cases, however, are unevenly distributed, with the highest proportion being reported in the North and Northeast regions of the country^3^. In 2016, 262 deaths due to leishmaniasis were recorded in Brazil (lethality: 9.0%), with the majority of deaths occurring in the Northeast (56.9%) and Southeast (26.7%) regions^4^.

Mortality due to leishmaniasis is considered to be potentially avoidable and is generally associated with malnutrition, late diagnosis, and HIV infection. Individuals with VL/HIV coinfection are three times more likely to die than the general population, and the lethality rate may reach 25% in coinfected individuals^5^. However, at the community level, other factors may be associated with the high disease burden and culminate in deaths. In this sense, the living conditions of the population, so-called Social Determinants of Health (SDH), have been associated with the maintenance of the leishmaniasis transmission chain in low - and middle-income countries. Thus, investigations addressing this issue have special relevance for public health, since they can provide suitable information to strategies.

The two widely used methods of measuring the level of population development and the degree of social vulnerability are as follows: a) Municipal Human Development Index (HDI), which measures the degree of development of municipalities in three dimensions (income, longevity, and education), and b) Social Vulnerability Index (SVI), which is used to determine the degree of vulnerability and social exclusion of municipalities in three domains (urban infrastructure, human capital, and income and work).

We aimed to analyze the relationship between the mortality rate due to visceral leishmaniasis and the indicators of human development and social vulnerability in Brazil.

We performed an ecological study of all visceral leishmaniasis deaths in Brazil, from 2001 to 2015. Brazilian regions, states, and their capitals were considered as units of analysis. Data on leishmaniasis deaths were obtained from the Mortality Information System (SIM, acronym in Portuguese) using the International Statistical Classification of Diseases and Related Health Problems (ICD-10). The ICD-10 code used to obtain leishmaniasis deaths was B55.0-visceral leishmaniasis, from the microdata of referred system (http://www2.datasus.gov.br/DATASUS/index.php?area=0901&item=1&acao=26&pad=31655). Absolute annual population was obtained from the Brazilian Institute of Geography and Statistics (IBGE, acronym in Portuguese)^6^. Mortality rate was calculated using the following equation: average deaths during the period/population of the middle of the period × 100,000 population.

We selected 49 social indicators of human development and social vulnerability and grouped them into 10 blocks, according to the theme of the indicator [Supplementary data (Table 1)]. These indicators were obtained from the Atlas of Social Vulnerability (http://ivs.ipea.gov.br/index.php/pt/) from the Institute of Applied Economic Research and the Atlas of municipal Human Development Index (http://atlasbrasil.org.br/2013/) from the IBGE based on the 2010 Brazilian Census.

For the analysis of social indicators, the classic regression model (ordinary least square) was used for each of the ten blocks of indicators. Next, the independence of residues was tested to evaluate the need for incorporation of a spatial regression model. If it is found, the Lagrange multiplier tests are used to define the spatial model: Spatial Lag Model (LAG) or Spatial Error Model (SEM). The LAG attributes to the response variable Y as the ignored spatial autocorrelation, while the SEM considers the spatial effects as a noise to be removed. The final quality of the models is assessed using the Akaike information criterion (AIC), Schwarz information criterion (BIC), log-likelihood function, coefficient of determination, and residue independence criteria^7^.

The GeoDa 1.8.10 (Center for Spatial Data Science, University of Chicago, Chicago, Illinois, IL, USA) was used to analyze the association between mortality rates and social indicators. Maps were made using QGis software version 2.14.11 (Open Source Geospatial Foundation, Beaverton, Oregon, OR, USA).

This study did not require authorization from the Ethics Committee, as we used open public domain data without identifying individuals.

Between 2001 and 2015, a total of 4,158 visceral leishmaniasis-related deaths were recorded in Brazil, resulting in a mortality rate of 0.15/100,000 population. The highest mortality rates were reported in the Northeast region (0.30/100,000 population), followed by the North (0.26/100,000 population) and Central-West (0.25/100,000 population) regions. The lowest rates were reported in the South and Southeast regions. The South region recorded the lowest leishmaniasis mortality rate (<0.01/100,000 population) (Figure 1).

**FIGURE 1:**
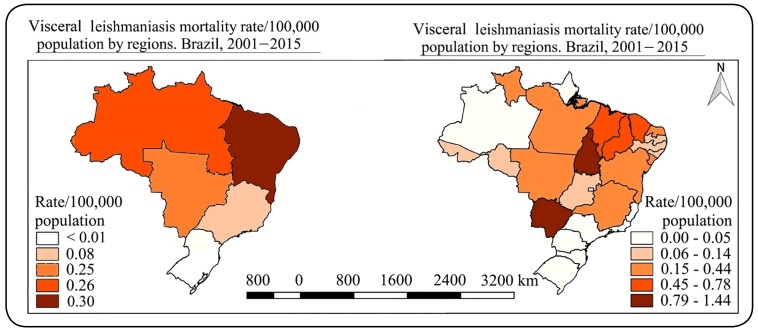
Spatial distribution mortality rate due to leishmaniasis in Brazil and its regions and states, 2001-2015.

When we analyzed the data of each Brazilian state, the highest leishmaniasis mortality rates were observed in Tocantins (271 deaths; mortality rate of 1.43/100,000 population), Mato Grosso do Sul (267 deaths; 0.77/100,000 population), Maranhão (635 deaths; 0.67/100,000 population), and Piauí (301 deaths; 0.65/100,000 population). 

Mortality rate analysis by capital showed high rates in the cities of Palmas/Tocantins (34 deaths; mortality rate: 1.27/100,000 population), Campo Grande/Mato Grosso do Sul (120 deaths; 1.09/100,000 population), and Teresina/Piauí (101 deaths; 0.88/100,000 population). Fortaleza/Ceará (n=167), Belo Horizonte/Minas Gerais (n=161), and Campo Grande/Mato Grosso do Sul (n=120) municipalities had the highest absolute numbers of deaths.

In the analysis by the OLS, 12 social indicators had an association with leishmaniasis mortality rate: one in the SVI Urban Infrastructure subdomain (% of the population living in urban households without the collection service of garbage), two in the SVI Human Capital subdomain (% of people aged 6-14 years of age who do not attend school and % of women aged 10-17 who had children), one in the SVI Income and Work subdomain (population unemployment rate in those aged ≥18 years), one indicator on HDI income (income per capita), and seven in block 10 (illiteracy rate for people aged ≥18 years and ≥25 years; Gini index; % of employed persons ≥18 years old; % of employed persons aged ≥18 years who completed the fundamental school; % of employed persons aged ≥18 years who completed high school; average income of employed persons aged ≥18 years) [Supplementary data (Table 2)].

Residues of the classical regression model did not present spatial dependence in the Moran statistic; for this reason, the spatial regression was not applied.

Visceral leishmaniasis remains an important public health concern in Brazil. Our findings revealed high leishmaniasis mortality rates throughout the country, especially in the North and Northeast regions. These findings may be due to the following reasons: the disordered process of urbanization, with consequent precarious housing conditions close to places where both disease-transmitting vectors and reservoirs are present; deficiencies in vector control; and late diagnosis, which compromises patient prognosis^8^ ^,^ ^9^.

The environmental and social settings experienced by populations in the North and Northeast regions are vulnerable to the spread of leishmaniasis^9^. Migration to urban centers can result in disorderly urbanization, without adequate infrastructure and with inadequate housing in peripheral areas near forests where both vectors and natural hosts are found. This creates a favorable epidemiological context for disease transmission^10^. This study found a strong relationship between high mortality by visceral leishmaniasis and indicators of social vulnerability, especially poor garbage collection, low schooling, unemployment, and low income.

The combination of these epidemiological factors resulted in the spread of leishmaniasis in Brazil, especially in previously non-endemic areas. A nationwide study in Brazil revealed that cases of leishmaniasis were recorded in 11.7% of municipalities in 2002, increasing to 16.8% in 2014^11^. These studies suggested that the main cause of the spread of leishmaniasis was the rural-to-urban migration process, since approximately 70% of cases registered between 2002 and 2014 occurred in individuals living in urban areas^11^. Migratory movement toward new agricultural centers in Brazil may also explain the high leishmaniasis mortality in Tocantins, Mato Grosso do Sul, and Goiás.

In Rio de Janeiro, studies have identified the following factors as the cause of leishmaniasis transmission and subsequent high mortality: i) the migratory process of the population, ii) environmental degradation with consequent vector density increase, iii) high population of infected animals making reservoir control difficult, and (iv) population living in subnormal settlements and with conditions of social vulnerability^11^. The territorial configuration and the mode of occupation of the geographic space are intrinsically related to the transmission of the disease in the state of Rio de Janeiro^12^.

In addition to the urbanization process^13^, it is important to consider the environmental context and climate change in relation to the spread and maintenance of leishmaniasis. Hot and humid climates favor vector proliferation, increasing the risk of transmission, especially in urban areas with population agglomeration^13^.

The stable national trend in mortality due to leishmaniasis and the increasing trend in some subnational units suggest failures in control programs as well as in the underlying health system. High seroprevalence of Leishmania infection in dogs often precedes high prevalence of leishmaniasis in humans. However, operational difficulties in identifying animal reservoirs allow maintenance of the transmission chain^14^. Furthermore, operational difficulties are also observed in the health system, mainly due to the difficulty of access to health services among poorer population groups, who are often at greater risks of neglected diseases, and due to the poor capacity of health services to identify cases of leishmaniasis early in the community and, consequently, to provide adequate management^13^ ^,^ ^14^.

Another important factor for the stable or even increasing trend of leishmaniasis mortality is the geographical overlap of leishmaniasis with major areas of HIV transmission^15^, since patients coinfected with HIV are at higher risk of death than non-coinfected patients^3^ ^,^ ^5^. Research conducted in Minas Gerais showed that the lethality due to leishmaniasis was 1.9 higher in HIV+ individuals, with an odds ratio (OR) of 10.9 and an unfavorable outcome up to 6 months after diagnosis^15^.

Notwithstanding all methodological precaution, this study has some limitations. The main limitation was the quality of death records, especially in small municipalities in the North and Northeast regions, which may suggest the existence of data underreporting. These localities often face structural problems in disease surveillance, resulting in inadequate death investigation. This process is perceived, for example, by the discrepancy between deaths recorded in SIM and the National Notifiable Diseases Information System (SINAN, acronym in Portuguese): deaths due to leishmaniasis registered in SIM were not recorded in the SINAN and not included in the death certificate. Despite these limitations, our findings and their interpretations are supported by 15 years of information on leishmaniasis mortality collected in Brazil.

In conclusion, the results showed that disparities in the distribution of income, poverty, low educational level, and fragile housing conditions were the determinants associated with higher leishmaniasis mortality in Brazil.

This indicates that control measures should be adopted and/or strengthened to reduce cases of leishmaniasis, including measures that facilitate early diagnosis and improve individuals’ access to health services resulting in better prognosis, with subsequent mortality reduction. We observe that the solution to this problem is complex and goes beyond patient diagnosis and treatment. It is necessary to implement comprehensive public policies capable of influencing social determinants of the disease, such as poor basic sanitation and undiagnosed street animals, which prolong the transmission chain.

## References

[1] World Health Organization (WHO) (2018). Global leishmaniasis surveillance update,
1998-2016. Wkly Epidemiol Rec.

[2] Organização Pan-Americana Saúde (OPAS) (2018). Leishmanioses: Informe Epidemiológico das Américas.

[3] Lindoso JA, Cota GF, Cruz AM, Goto H, Maia-Elkhoury AN, Romero GA (2014). Visceral Leishmaniasis and HIV Coinfection in Latin
America. PLoS Negl Trop Dis.

[4] Ministério da Saúde (MS). Secretaria de Vigilância em Saúde (2017). Letalidade de Leishmaniose Visceral. Brasil, Grandes Regiões e Unidades
Federadas. 2000 a 2013..

[5] Sousa-Gomes ML, Romero GAS, Werneck GL (2017). Visceral leishmaniasis and HIV/AIDS in Brazil: Are we aware
enough?. PLoS Negl Trop Dis.

[6] Instituto Brasileiro de Geografia e Estatistíca (IBGE) (2010). Censo Demográfco 2010: Características urbanísticas do entorno dos
domicílios.

[7] Anselin L, Florax RJ, Anselin L, Florax RJ (1995). Small sample properties of tests for spatial dependence in
regression models: Some further results. New Directions in Spatial Econometrics.

[8] Toledo CRS, Almeida AS, Chaves SAM, Sabroza PC, Toledo LM, Caldas JP (2017). Vulnerabilidade à transmissão da leishmaniose visceral humana em
área urbana brasileira. Rev Saude Publica.

[9] Martins-Melo FR, Lima MS, Ramos AN, Alencar CH, Heukelbach J (2014). Mortality and case fatality due to visceral leishmaniasis in
Brazil: a nationwide analysis of epidemiology, trends and spatial
patterns. PLoS ONE.

[10] Nguyen LD, Raabe K, Grote U (2015). Rural-Urban Migration, Household Vulnerability, and Welfare in
Vietnam. World Dev.

[11] Reis LL, Balieiro AAS, Fonseca FR, Gonçalves MJF (2017). Changes in the epidemiology of visceral leishmaniasis in Brazil
from 2001 to 2014. Rev Soc Bras Med Trop.

[12] Kawa H, Sabroza PC, Oliveira RM, Barcellos C (2010). A produção do lugar de transmissão da leishmaniose tegumentar: o
caso da Localidade Pau da Fome na cidade do Rio de Janeiro,
Brasil. Cad. Saúde. Pública.

[13] Diniz LFB, Souza CDF, Carmo RF (2018). Epidemiology of human visceral leishmaniasis in the urban centers
of the lower-middle São Francisco Valley, Brazilian semiarid
region. Rev Soc Bras Med Trop.

[14] Alves Eb, Figueiredo Fb, Rocha Mf, Werneck Gl (2018). Dificuldades operacionais no uso de coleiras caninas impregnadas
com inseticida para o controle da leishmaniose visceral, Montes Claros, MG,
2012. Epidemiol. Serv. Saúde.

[15] Cota GF, Sousa MR, Mendonça ALP, Patrocínio A, Assunção LS, Faria SR (2014). Leishmania-HIV Co-infection: Clinical Presentation and Outcomes
in an Urban Area in Brazil. PLoS Negl Trop Dis.

