# Supplementary material for: Social determinants of mortality due to visceral leishmaniasis in Brazil (2001-2015): an ecological study
Source: Rev Soc Bras Med Trop. 2019 Dec 20;53:e20190262. doi: 10.1590/0037-8682-0262-2019 (PMC7083365; doi:10.1590/0037-8682-0262-2019)
Supplement: Supplementary file 1 [file 1678-9849-rsbmt-53-e20190262-suppl1.pdf]

Rev Soc Bras Med Trop | on line | Vol.:53:e20190262, 2020

TABLE 1: Social determinants of health included in the data analysis (n=49).

**Indicators and blocks**
**Block 1 - Synthetic indicators of Social Vulnerability and Human Development**
Social Vulnerability Index (**SVI**)
Municipal Human Development Index (**MHDI**)
**Block 2 - Domains of the Social Vulnerability Index**
SVI Urban infrastructure
SVI Human capital
SVI Income and work
**Block 3 - Domains of the municipal Human Development Index**
MHDI Longevity
MHDI Education
MHDI Income
**Block 4 - SVI Urban infrastructure subdomains**
% of people in households with inadequate water supply and sewage
% of the population living in urban households without the garbage collection service
% of people living in households with a per capita income lower than half a minimum wage (from 2010) and spend more than an hour to go to work
**Block 5 - SVI Human capital subdomains**
Infant mortality
% of children aged 0-5 years who do not attend school
% of people aged 6-14 years who do not attend school
% of women aged 10-17 years who had children
% of mothers heads of household who did not complete the elementary education and with a child aged under 15 years
Illiteracy rate of the population aged ≥15 years
% of children living in households where none of the residents have completed elementary education
% of people aged 15-24 years who neither study nor work and have a per capita household income equal to or less than half a minimum wage (from 2010)
**Block 6 - SVI income and work subdomains**
Proportion of people with per capita household income equal to or less than half a minimum wage (from 2010)
Unemployment rate of the population aged ≥18 years
% of people aged ≥18 years who did not complete the elementary education and have no formal employment
% of people in households with per capita income less than half a minimum wage (from 2010) and are dependent of income from elderly
Occupancy rate of persons aged 10-14 years
**Block 7 - MHDI longevity subdomains**
Life expectancy at birth

CONTINUE....

**Block 8 - MHDI education subdomains**
Schooling sub index
% of people aged ≥18 years with completed elementary education
School attendance sub index
% of children aged 5-6 years who attend school
% of people aged 11-13 years who completed elementary education or are in last years of their elementary education
% of people aged 15-17 years who completed elementary education
% of people aged 18-20 years who completed high school
**Block 9 - MHDI Income subdomains**
Per capita income
**Block 10 - Other indicators of social vulnerability and human development**
Illiteracy rate of people aged ≥18 years
Illiteracy rate of people aged ≥25 years
Per capita income of people vulnerable to poverty
% of people who have income from work
Gini index
% of employed aged ≥18 years with formal work
% of employed aged ≥18 years without formal work
% of employed aged ≥18 years with formal work in public sector
% of self-employed aged ≥18 years
% of employers aged ≥18 years
Degree of formalization of the employed aged ≥18 years
% of employed people aged ≥18 years who completed elementary education
% of employed people aged ≥18 years who completed high school
% of employed people aged ≥18 years who graduated from college
Average income of employed persons aged ≥18 years
% of employed persons aged ≥18 years without income
