# Supplementary material for: Social determinants of mortality due to visceral leishmaniasis in Brazil (2001-2015): an ecological study
Source: Rev Soc Bras Med Trop. 2019 Dec 20;53:e20190262. doi: 10.1590/0037-8682-0262-2019 (PMC7083365; doi:10.1590/0037-8682-0262-2019)
Supplement: Supplementary file 2 [file 1678-9849-rsbmt-53-e20190262-suppl2.pdf]

TABLE 2: Social determinants of health associated with mortality due to leishmaniasis in the Brazilian states

Social determinants of health (SDH)	Coefficient	t-statistic	P-value
**Block 1 - Synthetic indicators of Social Vulnerability and Human Development**			
Constant	249.939	129.883	0.206
Social Vulnerability Index (**SVI**)	−0.7168	−0.55037	0.587
Municipal Human Development Index (**MHDI**)	−284.658	−131.204	0.201
**Block 2 - Domains of the Social Vulnerability Index**			
Constant	0.025052	0.084978	0.933
SVI urban infrastructure	−0.58397	−0.93874	0.357
SVI human capital	−0.03852	−0.02232	0.982
SVI income and work	113.209	0.703285	0.488
**Block 3 - Domains of the municipal Human Development Index**			
Constant	415.361	138.378	0.179
MHDI longevity	−519.888	−0.82902	0.415
MHDI education	483.948	182.823	0.080
MHDI income	−378.235	−115.753	0.258
**Block 4 - SVI Urban infrastructure subdomains**			
Constant	0.34427	226.467	0.033
% of people in households with inadequate water supply and sewage	−0.01409	−165.531	0.111
% of the population living in urban households without the garbage collection service	0.046009	277.332	0.010*
% of people living in households with a per capita income lower than half a minimum wage (from 2010) and spend more than an hour to go to work	−0.01797	−15.873	0.126
**Block 5 - SVI Human capital subdomains**			
Constant	−0.2469	−0.52858	0.603
Infant mortality	0.038386	137.215	0.186
% of children aged 0-5 years who do not attend school	−0.0009	−0.09345	0.926
% of people aged 6-14 years who do not attend school	−0.17718	−359.938	0.002*
% of women aged 10-17 years who had children	0.529425	41.596	0.000*
% of mothers who are heads of the household who did not finish elementary education and with a child aged below 15 years	−0.04108	−199.257	0.061
Illiteracy rate of the population aged ≥15 years	0.015218	0.554358	0.586
% of children living in households where none of the residents have completed elementary education	−0.01713	−11.598	0.261
% of people aged 15 to 24 who neither study nor work and have a per capita household income equal to or less than half a minimum wage (from 2010)	−0.00433	−0.15139	0.881

CONTINUE....

**Block 6 - SVI Income and Work subdomains**			
Constant	151.792	25.628	0.018
Proportion of people with per capita household income equal to or less than half a minimum wage (from 2010)	0.026026	154.767	0.136
Unemployment rate of the population aged ≥18 years	0.15252	−29.546	0.007*
% of people aged ≥18 years who did not complete elementary education and have no formal employment	−0.02451	−11.801	0.251
% of people in households with per capita income less than half a minimum wage (from 2010) and are dependent of income from elderly	0.128724	0.78679	0.440
Occupancy rate of persons aged 10-14 years	−0.06515	−132.226	0.200
**Block 7 - MHDI longevity subdomains**			
Constant	464.927	192.048	0.066
Life expectancy at birth	−0.05998	−182.203	0.080
**Block 8 - MHDI education subdomains**			
Constant	−485.388	−131.447	0.204
Schooling sub index	127.915	0.504881	0.619
% of people aged ≥18 years with completed elementary education	−126.651	−0.49846	0.623
School attendance sub index	203.811	0.766518	0.452
% of children aged 5-6 years who attend school	−0.49636	−0.74839	0.463
% of people aged 11-13 years who completed elementary education or are in last years of the elementary education	−0.45384	−0.7035	0.490
% of people aged 15-17 years who completed elementary education	−0.48807	−0.73109	0.473
% of people aged 18-20 years who completed high school	−0.5771	−0.84299	0.409
**Block 9 - MHDI income subdomains**			
Constant	0.489711	311.116	0.004
Per capita income	−0.00036	−171.522	0.048*
**Block 10 - Other indicators on social vulnerability and human development**			
Constant	−874.504	−0.42407	0.680
Illiteracy rate of people aged ≥18 years	154.751	−349.353	0.005*
Illiteracy rate of people aged ≥25 years	129.939	353.438	0.005*
Per capita income of people vulnerable to poverty	0.024644	163.518	0.133
% of people who have income from work	−0.01423	−0.44997	0.662
Gini index	138.591	246.624	0.033*
% of employed aged ≥18 years with formal work	0.092662	0.44399	0.666
% of employed aged ≥18 years without formal work	−0.05158	−0.26604	0.795
% of employed aged ≥18 years with formal work in public sector	0.165275	0.772904	0.457
% of self-employed aged ≥18 years	−0.06876	−0.34904	0.734
% of employers aged ≥18 years	0.780477	264.799	0.024*
Degree of formalization of the employed aged ≥18 years	−0.11439	−136.603	0.201
% of employed people aged ≥18 years who completed elementary education	−0.157882	227.542	0.046*
% of employed people aged ≥18 years who completed high school	−0.17849	−273.257	0.021*
% of employed people aged ≥18 years who graduated from college	0.062241	0.93755	0.370
Average income of employed persons aged ≥18 years	−0.00168	−231.717	0.042*
% of employed persons aged ≥18 years without income	0.055523	0.325147	0.751

*Statistical significance

OPEN ACCESS
https://creativecommons.org/licenses/by/4.0/
